# Supplementary material for: ESBL-producing Klebsiella pneumoniae in a University hospital: Molecular features, diffusion of epidemic clones and evaluation of cross-transmission
Source: PLoS One. 2021 Mar 24;16(3):e0247875. doi: 10.1371/journal.pone.0247875 (PMC7990301; doi:10.1371/journal.pone.0247875)
Supplement: Attachment — Submitted filename: Response to Reviewers.docx [file pone.0247875.s001.docx]

Dear Editorial Board Member,

We thank you for the review of our manuscript “ESBL-producing *Klebsiella pneumoniae* in a University hospital: Molecular features, diffusion of epidemic clones and evaluation of cross-transmission”. We are very grateful for the opportunity to address the reviewer concerns and comments, as well as some you raised. Below are our responses to these comments and questions. We hope that these responses address all of these comments, and we thank you and the reviewers for helping us to improve the quality of the manuscript.

**Response to the reviewer**

**Reviewer #1:**

***Comment 1*: Introduction is to short and must be extend for more information about current global and France situation with the *K. pneumoniae*.**

*Answer*: Additional informations has been added in the 'introduction' section (lines 63-65 and lines 70-71).

***Comment 2*: There is a lot of percent data but there is lack of statistics. It requires at least confidence interval and p value.**

*Answer*: We thank you for this advice but they are not applicable to our data. Although our study include many percent data these are only descriptive data without value comparisons or trend analysis. Our objective was only to describe the *K. pneumoniae* producing extended-spectrum β-lactamases population structure but not to present the evolution of this population over the study period.

***Comment 3*: In case of minor errors I notice: Line 26: Do not Begin abstract with the word “Purpose”. Line 40: do not use word “conclusion” twice in the one line.**

*Answer*: We agree with this comment. In the abstract, we have removed the paragraph headings (lines 26, 31 and 40).

***Comment 4*: Line 56: such as? Give some examples.**

*Answer*: Done line 56.

We hope that these responses address all of these comments, and we thank you and the reviewers for helping us to improve the quality of the manuscript.

Thank you again for your consideration,

Best regards,

Marlène
